# Spatial variations in urban woodland cooling between background climates

**DOI:** 10.1038/s41598-024-85059-8

**Published:** 2025-01-25

**Authors:** Liu J, Dennis M, Lindley S. J

**Affiliations:** Department of Geography, School of Environment, Education and Development, The University of Manchester, Arthur Lewis Building, Oxford Road, Manchester, M13 9PL UK

**Keywords:** LST, Landscape metrics, Urban climate, Adaptation, UHI effect, Vegetation, Environmental impact, Climate sciences, Ecosystem services, Urban ecology

## Abstract

Urban woodland composition and configuration have strong associations with land surface temperatures (LST), but the evidence is contradictory due to different spatial scales, regional climate zones, woodland types and urban contexts. In this study, we analyse associations between urban woodland and LST within and between five cities in different Köppen climate zones. Our consistent methodology is framed around local climate zones and conducted at a fine spatial scale. We find that urban woodland fragmentation, connectedness, and shape complexity all influence LST, though much less than overall cover. The importance of cover holds for all climates except for hot-desert (Cairo). Otherwise, every 1% increase in woodland cover corresponds to a reduction of LST of around 0.07 °C to 0.02 °C (London-*Cfb* > Toronto-*Dfa* > Nanjing-*Cfa* > Shenyang-*Dwa*). Within cities, increasing urban woodland cover generally reduces LST more in built-up compared to vegetated zones. Nevertheless, associations between local LST and urban woodland composition and configuration are highly heterogeneous across cities, especially in cooler climates. Thus, to unravel the complexities of urban woodland cooling, systematic analysis of contemporaneous local and regional factors is required.

## Introduction

The Urban Heat Island (UHI) effect refers to the phenomenon where temperatures within urban areas are elevated compared to their surroundings^1–3^. The UHI effect has many impacts on the urban environment and human well-being. For instance, during heat waves, it can increase water consumption and energy use^3,4^, reduce the liveability and comfort of cities^5,6^, and increase morbidity and mortality^7,8^. Urban Green Space (UGS) has been widely studied as a measure to mitigate the UHI effect^9,10^, as it is cost-effective, environmentally benign, and politically acceptable^11,12^. UGS refers to all types of vegetation cover and natural elements in the urban area^11,13^, such as urban parks, lawns, woodlands, and green roofs^10,12^. However, UGS is generally recognised as a whole entity instead of analysing the cooling effect of different UGS components^14^. Specifically, urban woodland, as one of the components of UGS, has an overall stronger cooling effect than other individual components, such as grassland^4,9,15^. The enhanced cooling is partly due to higher evapotranspiration rates from trees and partly due to higher shading^4,9^. Studies centred on UGS alone do not acknowledge the specific cooling effect of urban woodland and may underestimate its cooling capability relative to grassland. Thus, there is a need to study the specific effect of urban woodland on the urban thermal environment.

City-scale analyses of UGS cooling often rely on remotely sensed LST data and earth observation data. The spatial characteristics of UGS, including its composition and configuration (generally estimated by Landscape Metrics (LMs)), have been identified as having a significant influence on cooling, for instance, the percentage of landscape^12,16,17^, patch density^12,17^, edge density^16,17^, and shape-related metrics^10,12^. The composition describes the proportion and abundance^12,13,18^, while configuration refers to the physical arrangement, distribution, geometric complexity, connectivity, and degree of fragmentation^9,19,20^. However, previous studies show inconsistent results on the association between LMs and LST^9,12,17^. Firstly, although the negative correlation between the composition of urban woodland and LST is unequivocal^21–23^, it has been reported that the composition was more important associated with LST than the configuration in Shijiazhuang, China^9^, the Gwynns Falls watershed, USA^16^, and Baltimore, USA^17^, while the opposite was identified in Sacramento, USA^17^. Secondly, previous studies also highlighted that there has yet to be a consensus on how– or if—the spatial configuration of urban woodland affects its cooling effect^12,14^. For example, some studies identified significant correlations between LST and configuration metrics^17,18^, while others reported non-significant associations in Kaula Lumpur and Hong Kong^12^. Thirdly, both positive and negative correlations between some configuration metrics and LST were identified in previous studies. Specifically, negative correlations between urban woodland edge density and LST were reported in Gwynns Falls watershed, USA^16^, Baltimore and Boston, USA^21^, and Shanghai, China^22^, but the reverse was demonstrated in Beijing^23^ and Wuhan^24^. Patch density negatively correlated with LST in Shanghai^22^, while a positive correlation was found in Shijiazhuang^9^ and Beijing, China^23^. It was found that the mean patch area was positively correlated with LST in Baltimore but negatively correlated with LST in Sacramento^17^. Previous studies concluded that these contradictory reports may be due to differences in regional climate^9,15,17^, methods used^12,17^, urban form^15^, understorey characteristics^25^, and study scale^12,17^. Thus, it is vital to consider these factors when studying the complex association between LMs of urban woodland and LST.

Regional climate is one of the significant factors influencing the cooling effect of urban woodland^15^. Climatic factors such as rainfall and solar radiation vary significantly in different climatic regions^6,26^, which affect the shading and evapotranspiration intensity of urban woodland^14,25,27^. Specifically, cooling effect is enhanced in wet and cool climates due to higher evapotranspiration rates^26^, while high temperature and dryness reduce evapotranspiration and thereby limit cooling^14,28^. However, the effect of spatial patterns of urban woodland on its cooling effect is less understood due to the lack of comparison across climate zones^9,10,27^, which leads to inconsistent and even contradictory results^4,26,29^. Thus, climate-zone-based studies must be carried out to get a comprehensive and universal understanding of the cooling effect of urban woodland from the city level to a global scale^10,15^.

The local context is another factor influencing the association between urban woodland characteristics and LST. Nevertheless, many existing studies are aspatial without considering how associations vary at finer spatial resolutions or between local neighbourhoods^30^. The existing literature shows that urban woodland composition and configuration have varied effects on LST according to neighbourhood type^31^. For example, the degree of shading from urban woodland depends on surrounding land covers and structures^4,31^. Meanwhile, it was reported that the cooling of trees varies between cities and rural areas^14^. A 0 to 0.2 °C temperature reduction associated with additional trees was also found in most neighbourhoods, while it may cause an increase in temperature in compact midrise areas^32^. One study also identified a tenfold higher evapotranspiration rate for trees planted over grass than trees planted in paved tree pits but a much stronger cooling effect from shading from trees over asphalt than grass^25^. Accordingly, it has been recommended that future studies combine spatial analysis and typical analytical frameworks, such as the Local Climate Zone (LCZ) scheme, to better understand the spatially varied cooling effect of urban woodland^14,30^.

The LCZ scheme classifies urban landscape into 17 categories based on ten parameters related to structural and land cover properties that influence the thermal environment. Each class is taken to be distinctive in terms of building morphology, surface cover, materials, and human activity^33^. LCZs are widely employed to study the UHI effect, urban forms, and local thermal conditions^34^. Many studies identified the heterogeneity of LST across LCZs^31,34^, and the implications this has for the thermal behaviours of land cover types^34^. For example, although more coverage of urban woodland is associated with lower LSTs, the efficiency of this measure across LCZs remains to be determined^32^. Furthermore, LCZ typologies may infer different understorey characteristics, for instance, a higher propensity for trees over asphalt in the urban core (e.g. LCZ1 or 2) compared to trees over grass in lower density LCZs (e.g. LCZ5 or 6). Therefore, the LCZ scheme divides urban areas into zones with comparable thermal environments according to their local urban contexts. Compared to previous studies that analysed the association between spatial composition and configuration of UGS and LST^9,10,14^, employing the LCZ scheme as a spatial framework can enhance understanding of the locally and spatially varied thermal benefits of woodland in urban areas^14,31^.

Based on the above, understanding the effect of urban woodland spatial characteristics on cooling is being hampered due to the different methodologies and study scales used^12,17^, lack of analysis of spatial variations according to the heterogeneity of the local context^30^, and the lack of comparison across regional climates^13,27,28^. In this paper, we aim to understand how the composition and configuration of urban woodland influence LST across local and regional climate zones. We use landscape metrics to represent urban woodland spatial composition and configuration characteristics and analyse associations between these metrics and LST. The same fine-scaled spatial regression methods combined with the LCZ scheme have been used to analyse five cities, each in a different Köppen climate zone. This consistent approach avoids the influence of the different methodologies and study scales in previous studies, explicitly handles local spatial variation within LCZs, and takes account of different regional climates. The study addresses three key research questions: (1) How do fine-scale associations between LST and urban woodland characteristics vary between cities in different Köppen climate zones? (2) To what extent do the observed LST and urban woodland associations spatially vary within cities? (3) Which urban woodland composition and configuration characteristics are associated with the lowest LSTs, taking account of local and regional climate zones? By answering these questions, we identify similarities and differences between climate zones and some of the reasons for inconsistent results in previous research.

## Results

### The thermal environment of the cities

**Fig. 1:**
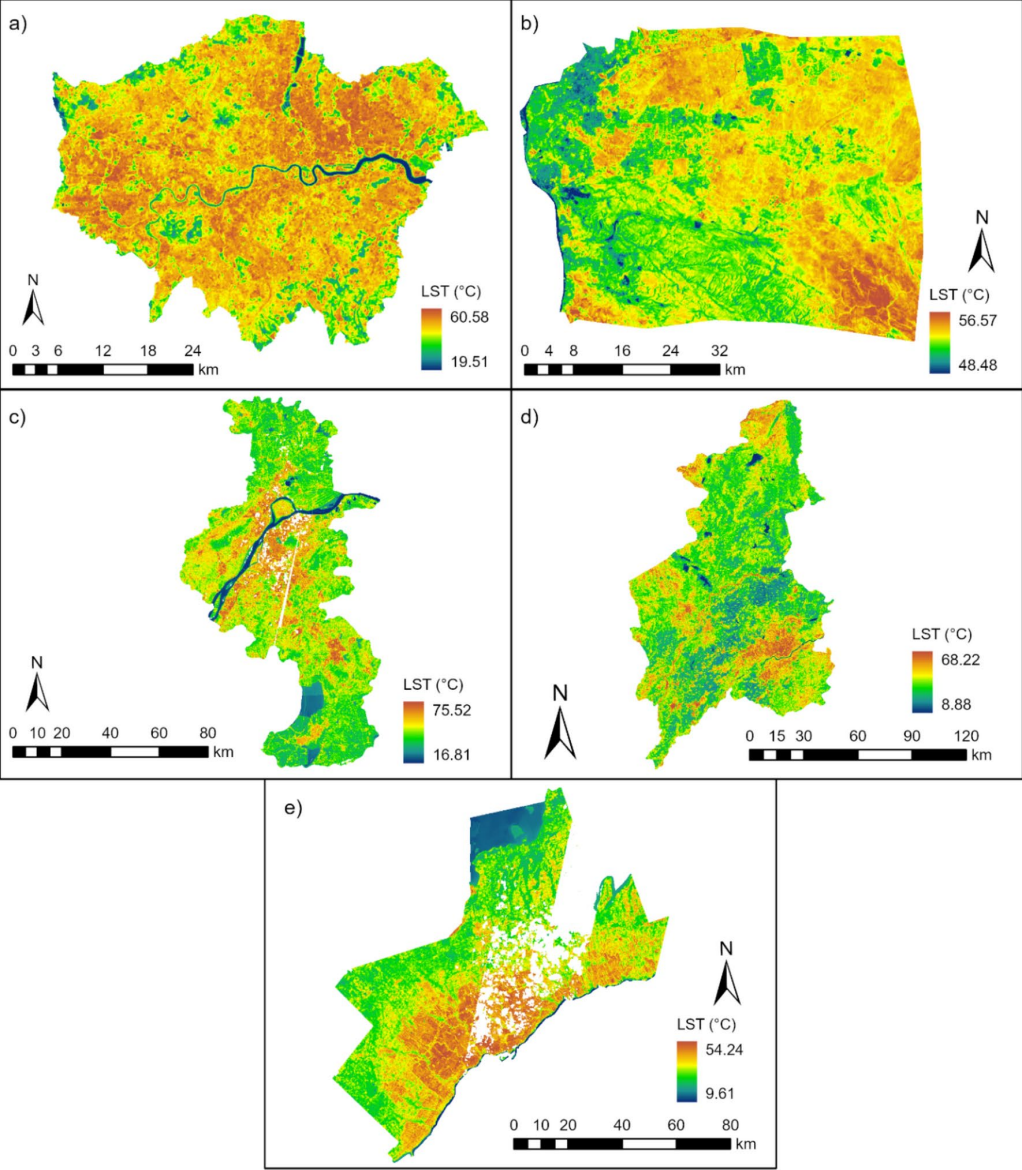
LST distribution of the cities. (**a**–**e**) presents the LST in Greater London, Greater Cairo, Nanjing, Shenyang, and Greater Toronto, respectively. The gaps within Nanjing and Greater Toronto are the null values. ArcGIS Pro, Version 3.0.2 (https://www.esri.com/en-us/arcgis/products/arcgis-pro/overview).

Greater Cairo has the highest mean LST at 53.36 °C, followed by Nanjing (40.53 °C), Greater London (40.49 °C), Greater Toronto (36.48 °C) and Shenyang (31.67 °C) (Fig. 1). Greater Cairo also shows the least spatial variability (σ = 0.53) of the five cities. Water surfaces (LCZ G) have the lowest LST (Supplementary Fig. S3), with statistically significant cool spots located along the River Thames in Greater London, the Yangtze River in Nanjing, and the southern fringe of Greater Toronto (Supplementary Fig. S4). Comparatively high LSTs are identified in built-up (LCZ 1-6), compared to vegetated areas (LCZ A, B, and D), with ranges shown in Supplementary Fig. S3. As expected, statistically significant LST hot spots are more concentrated in urban areas than surrounding vegetated areas (Supplementary Figs. S3, S4), except in Greater Cairo.

**Fig. 2:**
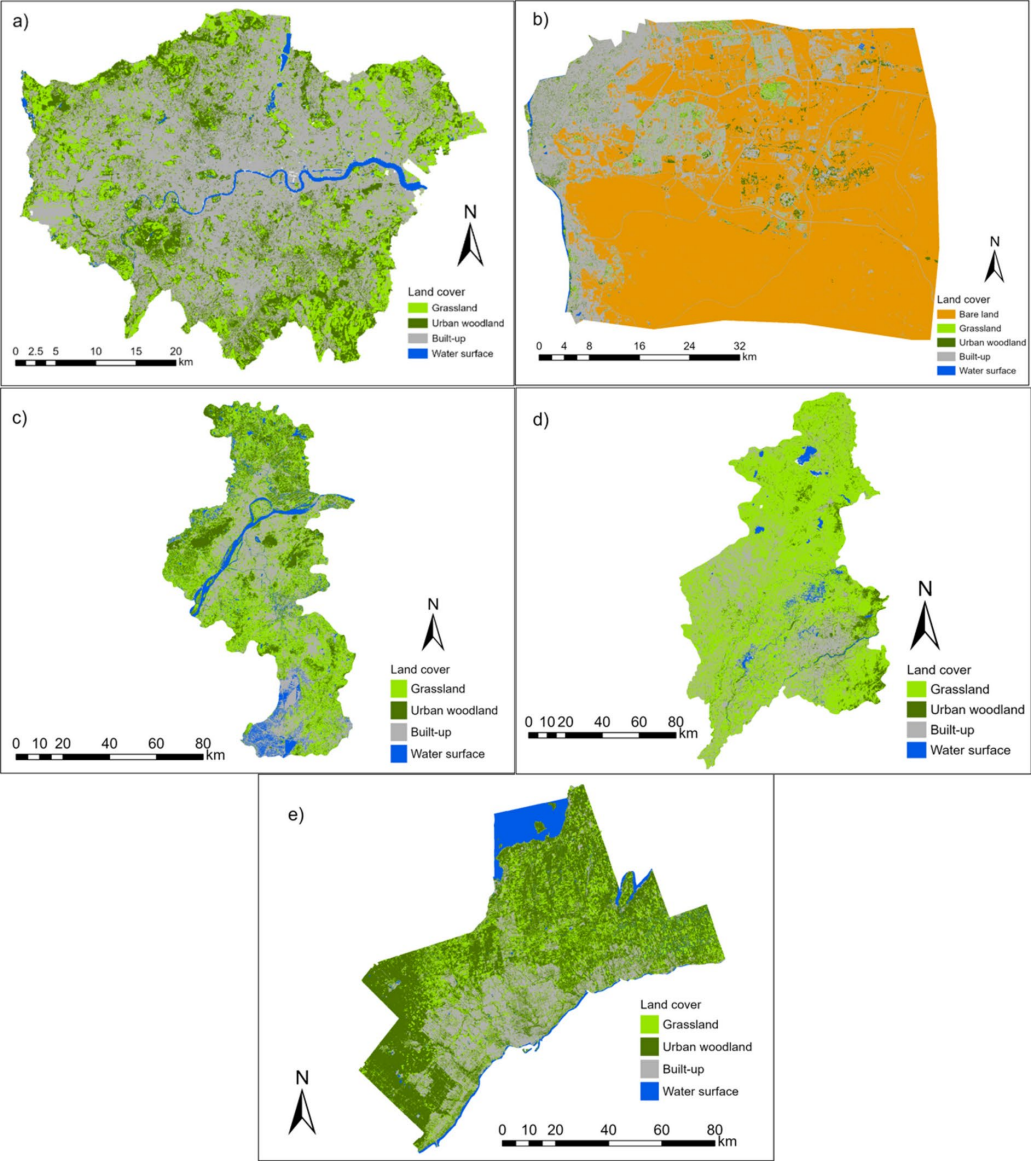
Land cover distribution of the cities. (**a**–**e**) presents Greater London, Greater Cairo, Nanjing, Shenyang, and Greater Toronto, respectively. ArcGIS Pro, Version 3.0.2 (https://www.esri.com/en-us/arcgis/products/arcgis-pro/overview).

The land cover also varies considerably (Fig. 2). Greater Cairo is dominated by bare land (Supplementary Fig. S5), and the limited vegetation coverage (5%) is concentrated along the River Nile (Fig. 2). In contrast, around 42% of Greater London is vegetated (Supplementary Fig. S5), with a more even distribution and larger patches towards the urban fringe (Fig. 2). In the other cities, > 60% of the land is vegetated, with Nanjing and Shenyang grassland-dominated and Greater Toronto woodland-dominated (Supplementary Fig. S5). These variations in land cover are partly climate-driven and may lead to different thermal environments.

For urban woodland, the highest woodland coverage is identified in LCZA (Dense trees), with cover ranging from 34.01% (Greater Cairo) to 75.64% (Greater Toronto) (Supplementary Fig. S6). Urban woodland configuration metrics also vary between cities. Taking edge density (ED) as an example (Supplementary Fig. S6), for nearly all the LCZ classes, the lowest ED range (90–190 m/hectare) is found in Greater London and the highest (160–240 m/hectare) in Greater Toronto. Therefore, urban woodland in Greater London is more aggregated and in Greater Toronto it is more fragmented.

### Model performance

The multi-linear regression models revealed that all 6 LMs at the 150 m study scale significantly correlate with mean LST (p-value < 0.001) in all the cities. Global OLS model R^2^ values show that OLS regression does not explain the association between LMs of urban woodland and LST as well as the local GWR models, and OLS is unreliable due to significant spatial autocorrelation identified by Moran’s I (Table 1). There is no widely accepted measure of statistical significance of GWR models. However, all the LMs of urban woodland are significantly correlated with LST based on a two-sided *t*-test at 95% confidence (Adjusted critical value of Pseudo-*t* Statistics > 1.96). GWR model performance is best in Greater Toronto, where LMs of urban woodland can explain 89% of the LST variation when considering local variability. Model performance is also good in Greater London and Greater Cairo (R^2^ at 0.84 and 0.81 respectively). It is less good in Nanjing and Shenyang but still acceptable, with R^2^ at around 0.80. Therefore, further analysis is based on the GWR model results.

**Table 1:** The performance of OLS model, GWR model and Moran’s I of the case studies.

	Greater London	Greater Cairo	Nanjing	Shenyang	Greater Toronto
R^2^ of OLS	0.56	0.16	0.12	0.04	0.39
σ^2^ of OLS	4.24	0.29	9.88	9.38	10.35
Moran’s I	0.51	0.42	0.54	0.65	0.57
R^2^ of GWR	0.84	0.81	0.79	0.80	0.89
σ^2^ of GWR	1.78	0.07	2.70	2.33	2.15

### The effect of urban woodland composition and configuration on LST

**Fig. 3:**
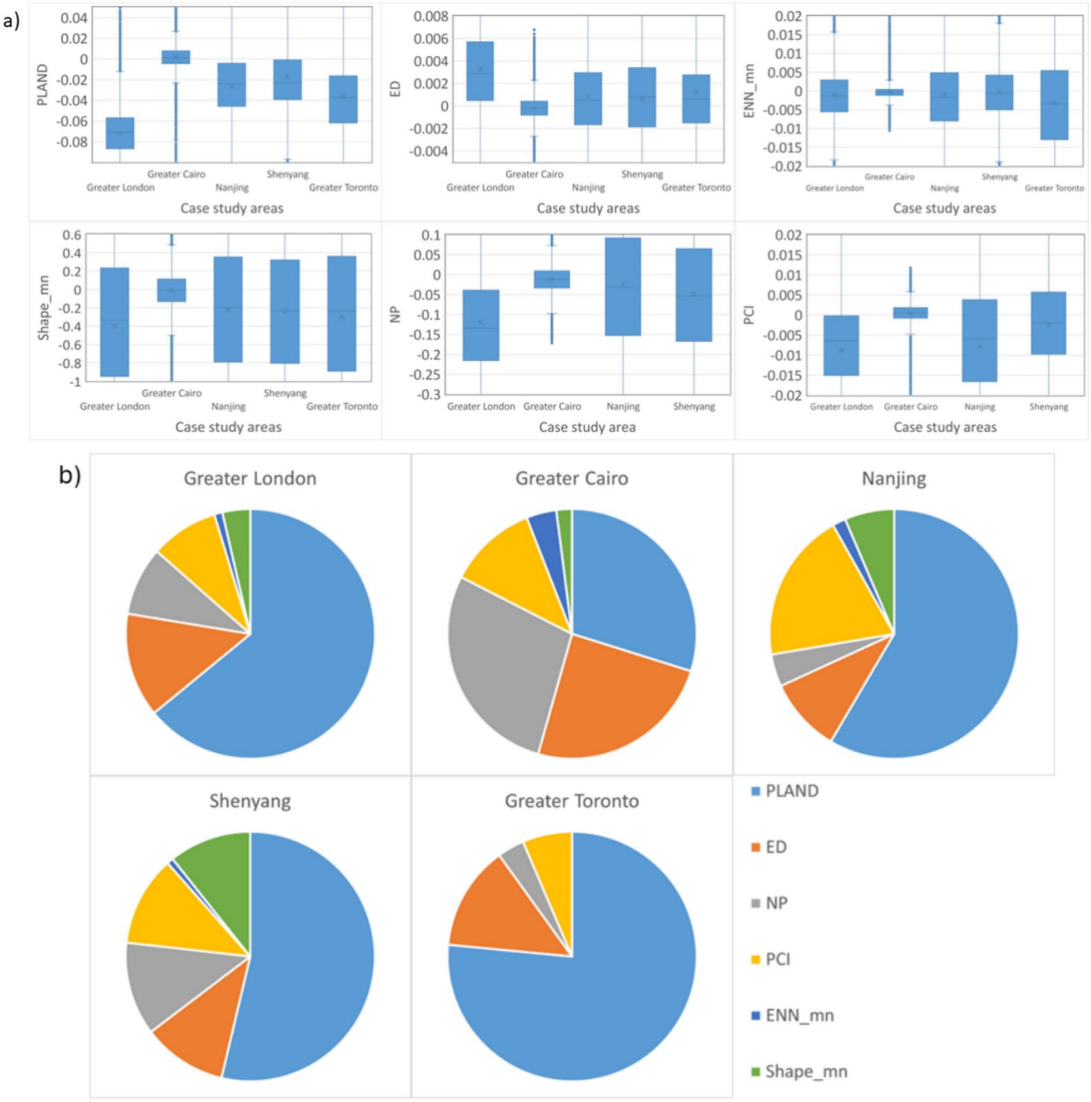
The effect of LMs on mean LST by city. (**a**) The coefficients from GWR model (PCI, NP, and Shape_mn area not included in the GWR model of Greater Toronto due to the local collinearity); (**b**) the relative proportions of standardised coefficients.

The GWR model coefficients (Fig. 3a and Supplementary Table S4) show that at the city scale, the effect of urban woodland LMs on LST varies between different regional climates. Specifically in Greater Cairo, the coefficients of all the LMs are close to 0 with the shortest ranges, indicating that the effect of spatial characteristics of urban woodland on LST is negligible. However, the coefficients are higher, varied between cities, and with more extensive ranges. The mean coefficients show that PLAND, PCI, NP, ENN_mn, and Shape_mn negatively correlate with LST, while ED correlates positively with LST (Fig. 3a and Supplementary Table S4). This suggests that LST decreases with increasing percentage coverage of urban woodland, and more aggregated, closer, and connected urban woodland patches with a more irregular shape. Specifically, a 1% increase in urban woodland coverage in Greater London is associated with LST reductions of ~ 0.07 °C, while less LST reduction was identified for the other cities, i.e. ~ 0.04 °C (Greater Toronto), ~ 0.03 °C (Nanjing), and ~ 0.02 °C (Shenyang). Similarly, the mean coefficient of ED in Greater London (0.0033) is higher than in the other cities (Supplementary Table S4), indicating that the edge density (degree of aggregation–fragmentation) of urban woodland affects LST more in Greater London than elsewhere. The box plots illustrate which metrics have the most uniform city-wide influence and which are most spatially heterogeneous, in some cases ranging from a relative cooling to a relative warming role depending on location. Taking PLAND as an example, the shorter range in Greater London indicates a more uniform cooling effect compared to Greater Toronto, where the larger range shows that the degree of associated cooling depends more on location (Fig. 3a and Supplementary Table S4). The spatial heterogeneity of effects is particularly marked for urban woodland complexity metrics across all cities. The influence of ED in Greater London is especially interesting, indicating a greater tendency towards a warming effect, which is heightened in more fragmented woodland and lessened in more aggregated ones.

The standardised coefficient (Fig. 3b and Supplementary Table S5) represents the relative overall influence of each LM on LST for each city. A key finding is that Greater Cairo differs from other cities. Here, PLAND, ED, and NP standardised coefficients have similar relative proportions, indicating that these LMs have a similar amount of influence on LST, albeit negligible and ranging from negative to positive (Fig. 3a, b). In the other cities, the composition metric PLAND has a much more substantial influence on LST than configuration metrics and has a predominantly negative effect (Fig. 3a, b). Figure 3b also shows how the influence of urban woodland spatial configurations on LST varies across cities. For example, ED is the most influential configuration metric in Greater London and Greater Toronto, compared to PCI in Nanjing (Fig. 3b and Supplementary Table S5).

### The varied effect between LCZ classes

**Fig. 4:**
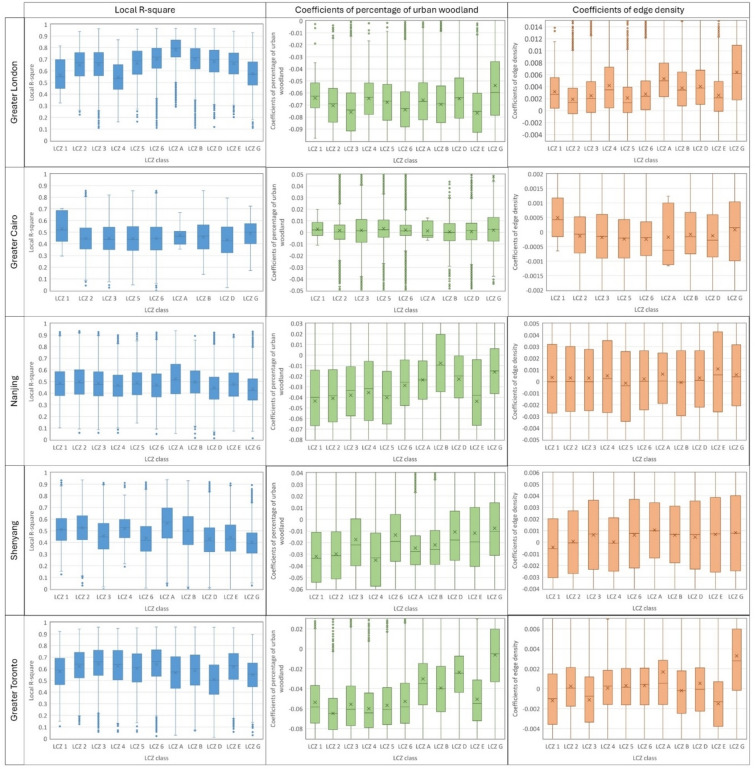
The local R^2^, coefficients of PLAND and ED between LCZ classes for each city.

The degree of spatial variability in the association of urban woodland composition and configuration on LST is analysed by combining the GWR model results and the LCZ classes. Firstly, the Local R^2^ values show where the LMs of urban woodland are more strongly associated with LST (Fig. 4). The Local R^2^ values of LCZs are most uniform in Greater Cairo and Nanjing (Fig. 4), indicating the degree of urban woodland LMs explaining LST variation is similar between LCZ classes. In contrast, more significant spatial variability is identified in the other cities (Fig. 4). Taking Greater London as an example, the LMs of urban woodland can explain ~ 80% of the LST variation in LCZ A (dense trees), though only 50% in LCZ 1, 4, and G (Compact high-rise, open high-rise, and water surface, respectively) (Fig. 4).

Secondly, the effect of urban woodland composition on LST varies spatially between LCZ classes in all the cities except for Greater Cairo. In Greater Cairo, the coefficient of PLAND is close to 0 across all LCZ classes (Fig. 4), indicating that changing urban woodland coverage has a negligible effect on LST regardless of LCZ class. Comparatively, in the other cities, the negative coefficient of PLAND is the lowest in LCZG (Water surface), identified in the Walthamstow Wetlands (Greater London) and along the Yangtze River (Nanjing) (Supplementary Fig. S7). Urban woodland coverage near water surfaces has a relatively small local cooling influence and, in some locations, is associated with warmer LST than the local surroundings. In contrast, a higher negative coefficient of PLAND is identified in built-up LCZ classes (LCZ1-6) than in vegetated LCZs (LCZ A, B, and D), especially in Nanjing, Shenyang and Greater Toronto (Fig. 4). Relatively low negative coefficients in vegetated LCZ classes are identified in urban parks and rural fringe areas, such as Richmond Park (Greater London), the northern part of Nanjing and Shenyang, and the western part of Greater Toronto (Supplementary Fig. S7). The different magnitudes of the negative coefficient indicate that urban woodland coverage in built-up LCZ classes generally has a more marked impact on LST. Within built-up LCZ classes (LCZ1-6), the magnitude of the negative coefficient varies between cities. For example, the higher negative coefficient is identified in LCZ3 and 6 in Greater London, while the opposite is seen in Shenyang (Fig. 4). Thus, LST reduction by increasing urban woodland coverage varies depending on the specific LCZ class and city.

Turning to urban woodland configuration, there is also a high degree of variability in the association with LST across LCZ classes in all cities except Greater Cairo. Taking ED as an example (Fig. 4), higher positive coefficients are identified in vegetated LCZs and LCZG, such as along River Thames and in Richmond Park (Greater London), along the Yangtze River and in Laoshan Forest Park (Nanjing), and the Terra Cotta conservation area and the southern fringe of Greater Toronto (Supplementary Fig. S8). These relatively high positive coefficients indicate a more aggregated distribution of urban woodland is associated with lower LST near the water surface and in vegetated areas, relative to a more fragmented distribution. In contrast, lower positive and negative coefficients are identified in built-up LCZs, such as LCZ1 in Shenyang and LCZ1 and 4 in Greater Toronto (Fig. 4). Negative coefficients are more likely to be identified near urban centres (Supplementary Fig. S8), indicating that a fragmented distribution of urban woodland is associated with lower LST in these areas.

**Fig. 5:**
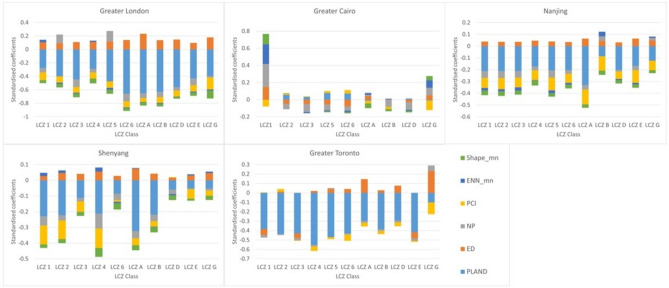
The standardised coefficients of GWR models between LCZ classes.

Lastly, the standardised coefficients represent the relative contribution of LMs on LST within each LCZ class among cities (Fig. 5). The highest standardised coefficient of PLAND means the composition of urban woodland contributes more to the LST variation than the other configuration metrics, with the exceptions of LCZ B in Nanjing, LCZ E in Shenyang, LCZG in Greater Toronto, and most of LCZ classes in Greater Cairo (Fig. 5). On the other hand, among all the configuration metrics, ED has the highest standardised coefficient in LCZ A in Greater London (0.23), Shenyang (0.07), and Nanjing (0.06) while it is lower in other LCZs (Fig. 5). The patch cohesion index has a high standardised coefficient in many LCZ classes in Nanjing and Shenyang (Fig. 5). These results indicate that the effectiveness of modifying urban woodland spatial composition and configuration varies depending on specific local contexts and cities.

## Discussion

This study quantifies the effect of urban woodland spatial composition and configuration on LST and how this effect varies spatially. The intra-city variation is quantified by combining GWR model results with the LCZ scheme, and the inter-city variation is identified by comparing cities in different climate regions. The results show that the composition and configuration of urban woodland significantly influence LST except in Greater Cairo, and the effect varies depending on the specific LCZ class and city. The findings shed light on the inconsistent results identified by the previous studies^12,17^ and provide practical implications for urban planning.

The results show that the urban woodland composition and configuration can explain more LST variation in cooler climates with medium humidity (Tables 1, 2), which is consistent with previous studies^9,17,27^. Specifically, urban woodland landscape metrics explain the highest variance of LST in Greater Toronto and Greater London, where there are moderate air temperatures and less extreme monthly precipitation (Tables 1, 2). Comparatively, the GWR models worse in Nanjing, Shenyang, and Greater Cairo than Greater London and Greater Toronto, which are hotter and/or have more extreme precipitation regimes. Moreover, a more substantial effect of urban woodland composition and configuration on LST is also identified in cooler climates, which is consistent with previous studies that varied regional climate conditions lead to these different effects^15,17,21,24^. In Greater Cairo’s hot and dry climate region, the effect of urban woodland composition and configuration on LST is negligible (Fig. 3a). This is likely due to environmental factors, such as water availability and soil moisture, which strongly influence the stomatal conductance of trees. Urban woodland cooling in Greater Cairo is, therefore, likely to be lower due to water stress and relatively low soil moisture, which limit urban woodland evapotranspiration^14,35^ and result in negligible latent heat flux from urban woodland^3^. In contrast, Nanjing is hot but humid, with a more substantial association between urban woodland composition and configuration and LST (Fig. 3a). This difference between Greater Cairo and Nanjing suggests that multiple strategies should be combined in hot and arid regions to improve the urban thermal environment, such as adding reflective surfaces and tree species with less irrigation requirement^9^.

**Table 2:** The climate of the case studies^44^, population and monthly average meteorological data during July over 5 years.

	Greater London	Greater Cario	Nanjing	Shenyang	Greater Toronto
Climate zone	Temperate oceanic climate (Cfb)	Hot desert climate (BWh)	Humid subtropical climate (Cfa)	Monsoon-influenced humid continental climate (Dwa)	Humid continental climate (Dfa & Dfb)
Characteristics of summer	Warm summers	Hot, dry and rainless	Very hot and muggy	Hot, humid summers	Warm or hot moderate to high humidity
Population (million)	8.9	22.18	8.51	8.29	5.93
Air temperature (°C)	19.5	30.36	28.46	26.08	22.22
Precipitation (mm)	19.5	< 1	167.92	90.18	76.66
Wind speed (km/h)	13.2	14.16	10.08	8.17	12.55

Despite the more substantial effect of urban woodland composition and configuration on LST found in cooler climate regions, the effectiveness varies. For instance, a per unit increase in woodland coverage may be most impactful on LST in Greater London (Fig. 3a). Modifying the spatial configuration of urban woodland provides different LST reductions between cities, such as increasing aggregation, which has a stronger influence on LST in Greater London than in the other cities, helping to offset the positive associations between high fragmentation and local LST. These findings underscore the need for context-specific strategies in urban planning and climate mitigation.

The land cover of each city may influence the effect of landscape composition and configuration of urban woodland on LST, and a more substantial effect is identified in cities covered by more urban woodland. Specifically, the lowest urban woodland coverage in Greater Cairo may be a reason of the negligible cooling effect, as small green spaces usually have a high thermal load and provide less cooling effects^10^. In contrast, the stronger association identified in Greater Toronto and Greater London may be due to the higher coverage of urban woodland (Supplementary Fig. S5) and the structural and biophysical properties of urban tree species^36^. Comparatively, Nanjing and Shenyang are mainly covered by grassland, and the humid conditions enhance the evaporation cooling of grassland in these two cities^9^. This may explain why the cooling effect of urban woodland is less prominent than that in Greater London and Greater Toronto.

Our results suggest that the fragmentation, connectedness, and shape complexity of urban woodland have some influence over LST, while their contributions matter less than the overall coverage of urban woodland for reducing LST in subtropical, temperate, and continental climate regions. Thus, introducing more urban woodland cover would be the most effective way to reduce LST. Comparatively, urban woodland composition contributes similarly to several configuration metrics in hot desert climates, such as Greater Cairo (Fig. 3b). In Greater Cairo, the high standardised coefficients of the number of patches indicate that adding patches of urban woodland would be the most effective in reducing LST, though the overall impact of urban woodland is minimal here. The result in Greater Cairo is consistent with previous studies that found the higher temperatures, low relative humidity, lack of water supply, and limited tree cover place a restriction on the cooling influence provided^9,27^. Due to limited evaporative cooling in hot desert climates, the shading effect from urban woodland is of higher importance^37^, which explains why the spatial configuration of urban woodland contributes more than its composition in drier climates^17^.

Our results demonstrate that the effect of urban woodland spatial composition and configuration on LST spatially varies more substantially in cooler climates than in hotter climates (Fig. 4). Specifically for Greater Cairo, the arid climate may limit the cooling effect of urban woodland, and the limited urban woodland coverage may be insufficient to cool the surface. The hot and humid climate in Nanjing limits the sensible heat flux, which may be the reason for the less spatial heterogeneity^21^. Therefore, in hot climates, the effect of urban woodland composition and configuration on LST tends to be more identical regardless of the local context.

The effect of urban woodland composition on LST spatially varied between LCZ classes in all the cities except for Greater Cairo, which means the cooling amounts provided by adding urban woodland coverage differ due to the surroundings. Near the water surface (LCZ G), the cooling effect of urban woodland is relatively small (Fig. 4), which is consistent with previous studies^38^. Compared with urban woodland, the water surface has lower LST, and it may exert a more substantial cooling effect, which may be the reason for the relatively lower cooling effect and even the warming effect of urban woodland near the water surface.

As might be expected, adding urban woodland coverage in built-up LCZs (LCZ 1–6) would generally result in more LST reduction than in vegetated LCZ classes (LCZA, B, and D) (Fig. 4). This result agrees with previous studies that urban woodland provides more cooling in areas covered by asphalt and impervious surfaces^25^. Moreover, compared with the other built-up LCZs, more urban woodland coverage in LCZ3 (Compact low-rise) and LCZ6 (Open low-rise) may provide a stronger cooling effect in Greater London. Similar results have been identified in previous studies, showing that the cooling effect of green space is better in low-rise and low-density urban settings than in high-rise, high-density areas^39^. However, this pattern does not hold for Shenyang and Nanjing, and to some extent Greater Toronto, possibly due to the different landscape characteristics.

We also found that the influence of woodland configuration on LST varies between LCZ classes (Fig. 4). For example, aggregated urban woodland is associated with relatively low LST in vegetated LCZ classes, especially in LCZA (Dense trees) and G (Water surface). In contrast, fragmented urban woodland in Shenyang and Greater Toronto tends to be associated with lower local LST in the most built-up LCZ classes. Fragmented urban woodland provides more shading^9,17^, which is more needed in built-up LCZ classes, while the aggregated distribution enhances evapotranspiration cooling^17^ and thereby better cools the surface in vegetated LCZs. Previous studies recommended reducing the fragmentation of urban woodland to enhance its cooling effectiveness as the evaporative cooling surpasses the shading effect^9^. However, our results suggest that the influence of fragmentation depends on specific locations, as the importance of evaporative cooling and shading effects varies spatially according to local context. For instance, the relative degree of evaporative cooling has been shown to be dependent on whether woodland is planted over grass or over paved surface^25^ and will also be influenced by water availability.

### Implications, recommendations, and limitations

Our results suggest that different strategies should be applied to promote the cooling effectiveness of urban woodland based on the specific regional climate and local context. Based on the results of this study, we have demonstrated that increasing urban woodland coverage would enhance cooling and help alleviate the UHI effect in most cities, except for those in hot and dry climates such as Greater Cairo (Fig. 3a). We have also shown that the spatial configuration of urban woodland is correlated with LST^7,10,18^. Our result shows that in general LST is lower where urban woodland patches that are larger, more aggregated, closer together, more connected and with more complex shapes, which is consistent with previous studies^10,18,20,30^. This is because the large and dense urban woodland is more heat tolerant^10^, aggregated and connected distribution enhances the evaporation cooling^17^, and the complex shape of urban woodland increases the shading effect. However, the effect of urban woodland composition and configuration varies in different LCZ classes.

Previous studies failed to generalize the conclusion because of the challenge of varying numbers and types of landscape metrics, changing spatial scales, and different study areas^12,23^. Our consistent methods facilitate comparison of the similarities and differences in the association between spatial characteristics of urban woodland and LST. Such comparisons will eventually lead to more insights about promoting the cooling effectiveness of urban woodland. Additionally, spatial regression method and landscape classification schemes, such as the GWR model and the LCZ scheme, are useful to overcome spatial autocorrelation when examining the association between spatial characteristics of urban woodland and LST. This helps understand the spatial variations and has been rarely considered in previous studies^12^.

Several factors have limited this study. Firstly, the study scale of 150 m was identified based on an analysis carried out for Greater London and is assumed to be appropriate for the other cities. However, there are no consistent results about which study scale is most suitable for analysing the association between landscape metrics and LST, and a different value may have been generated if one of the other cities had been assessed^40^. Simultaneously, the zoning problem of the modifiable areal unit problem (MAUP), referring to the variation in analytical results derived from how the data is zoned or grouped^41,42^, may influence the results after combining the LCZ classes. However, the zoning problem is closely related to the scale problem, which was thoroughly tested in the study, and set the foundation for addressing the zoning problem effectively^41^. In further studies, the zoning problem should be addressed by comparing and selecting the appropriate zoning approaches^41,42^, such as administrative and the LCZ scheme. Secondly, the LST and land cover maps are derived from a single satellite image sensed at different times and days. This may cause some inconsistencies between landscape characterisations and LST. For instance, LSTs in Greater London were influenced by an unusually prolonged hot-dry period spanning six weeks from June 2018^43^. Indeed, we hypothesise that the local warming effect of high edge density (increased fragmentation) in Greater London, may be related to these conditions and the time of imagery (mid-morning). Here, it is possible that reduced sky-view factor from tree canopies may have inhibited long-wave radiation losses from local surfaces which were unusually hot and dry. Even though satellite images for all cities were sensed in the same period of the year, and the landscape would not change too much, the different timestamps of images (Supplementary Table S8) may therefore also explain some of the variation observed both in this study and the wider literature. Further studies are recommended to combine more satellite images and time-series datasets, especially to control for time-of-day and antecedent conditions. Thirdly, this study only takes five cities representing different climatic conditions. Although our analysis benefits from an in-depth study of each city, it is recognised that a larger sample would enable a broader range of urban forms and woodland types to be considered. Lastly, this study does not account for the different cooling contributions provided by different tree species. Among regional climate and environmental conditions, tree species is also a factor influencing the evapotranspiration and shading effect of urban woodland^25^. Specifically, a higher tree growth rate allows high transpiration cooling, and leaf anatomical characteristics may influence reflection and transmission on tree canopies^25^. However, this study focuses on local-scale analysis of the association between urban woodland composition and configuration and LST, while tree species studies depend on micro-scale analysis. Further studies should seek to carry out micro-scale analysis on the influence of tree species on the thermal environment.

## Conclusion

This study compared the spatially varied association between urban woodland composition and configuration and land surface temperatures by applying an identical methodology to five cities in different climate zones. We found that the effect of landscape metrics of urban woodland exhibits intra- and inter-city variation, and the same landscape characteristics of urban woodland are associated with different influences on LST. We conclude that (1) more urban woodland coverage that is aggregated and connected, with complex shapes, tends to be associated with lower LST at the city scale in all the climate regions except for the hot desert climate. The same modification in landscape composition and configuration of urban woodland results in different amounts of LST reduction between cities, such as adding 1% of the urban woodland coverage reduces LST by around 0.07 °C in Greater London, followed by 0.04 °C in Greater Toronto, 0.03 °C in Nanjing, and 0.02 °C in Shenyang. This variation may be because of the regional climate and the landscape characteristics of each city. (2) The correlation between urban woodland composition and configuration and LST varies spatially, and more substantial variation is found in cooler climate regions. (3) The effect of landscape metrics of urban woodland on LST varies spatially depending on different LCZ classes. Specifically, as a UHI mitigation measure, more percentage coverage of urban woodland is generally recommended in built-up LCZs than in vegetated LCZs and near water surfaces. Aggregated urban woodland tends to perform cooling better in vegetated LCZs, while fragmented distribution tends to be associated with lower local surface temperatures in built-up LCZs. The finding holds especially true for Shenyang and Greater Toronto, and it is notable that in Greater London, more fragmentation was predominantly associated with warming. These results may be due to images being captured at different times-of day and we therefore recommend that time-of-day is routinely reported in similar studies. The results highlight that the application of spatial regression methods, such as the GWR model, is more appropriate for quantifying the association between landscape metrics of urban woodland and LST than OLS models. Therefore, to better mitigate the UHI effect, urban woodland with different spatial patterns should be implemented based on local context and regional climate to maximize its cooling effectiveness.

## Methodology

### Study area

**Fig. 6:**
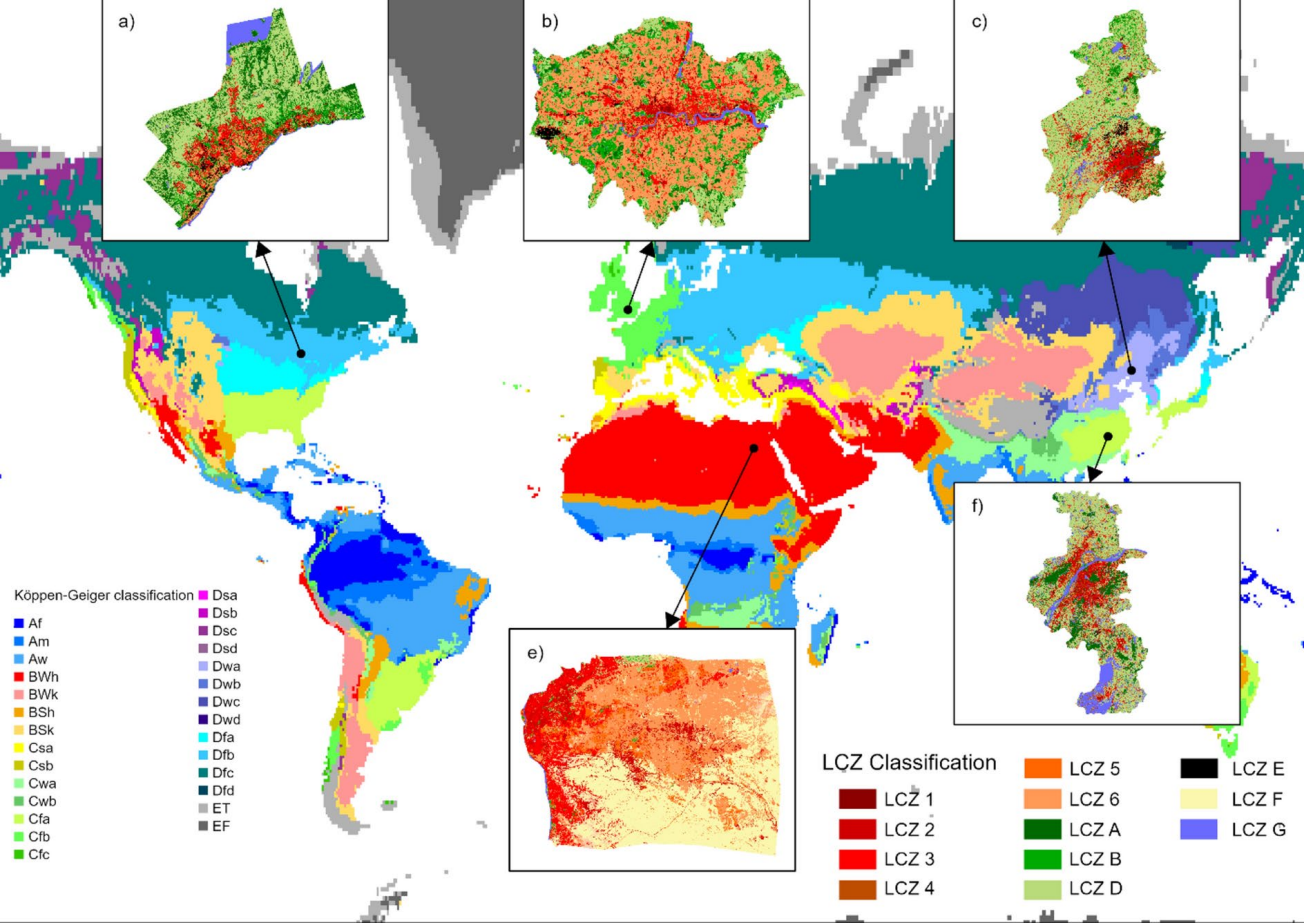
Distribution of the case study areas according to Köppen–Geiger climate classification^44^, this work is licensed under CC BY 4.0 and LCZ maps of each city (https://www.wudapt.org/).

Following the Köppen–Geiger climate classification scheme^44^, urban zones in different climate zones were filtered, and five case study areas were selected (Fig. 6). All five mega-cities cover a large, densely populated area, either already well-developed or undergoing rapid urbanization. The large area of the cities ensures sufficient land cover types with different landscape characteristics. The dense population and urbanization imply that the impervious surfaces essentially replace the natural cover of the case studies, and therefore, the cities are affected by the UHI effect. The five cities have distinct regional climate and meteorological parameters, including air temperature, precipitation, and wind speed (Table 2). They are located in heavily populated regions on different continents, including Europe, Africa, Asia, and North America. Additionally, the selected cities are in developed and developing countries, which avoids bias in selecting study areas only from the global north.

### Data collection and preprocessing

#### Land cover and Landscape metrics (LMs)

The land cover of each case study area was classified into four classes, including built-up area, urban woodland, grassland, and water surface. This classification distinguishes grassland from urban woodland, while water surface and built-up area influence the thermal environment. The urban woodland in this study refers to total tree canopy coverage located within the study areas, regardless of different tree species. The classification was based on the random forest model using the freely available Sentinel-2A, L1C dataset at 10 m resolution. The random forest model was trained by manually creating sample data, including over 300 sample points for each city. For each case study area, all the bands of Sentinel 2A data were used in the model for land cover classification. The random forest model performed well in classifying the land cover types (overall accuracy > 89% for all areas). High user and producer accuracy is also achieved for all the land cover types (Supplementary Table S6).

Based on the land cover classification map, LMs were calculated using the Landscape Metric package in R^45^. Class-level LMs were used in this study as they can describe the spatial composition and configuration of each land cover class. In contrast, landscape-level LMs rely on the mean value of the different classes, and patch-level LMs are average patches of all classes^40^. Six LMs with the least data redundancy and multicollinearity were identified as appropriate for analysing the association between urban woodland spatial composition and configuration and LST (See Supplementary files). The selected LMs were commonly analysed by previous studies and were attributed to cooling processes, easily retrieved and interpreted, and capable of characterising urban woodland spatial patterns^9,10,17,18^. Different study scales of LMs result in different correlation results^40^, but there is no general conclusion about the best study scale. The 150 m study scale was identified as appropriate for analysing the association between urban woodland spatial composition and configuration and LST (See Supplementary files). The selected LMs included one composition metric (PLAND) to depict the abundance of urban woodland and five configuration metrics, which were used to characterise the urban woodland in terms of its connectivity (PCI), shape complexity (Shape_mn), and aggregation or fragmentation (ED, ENN_mn, and NP)^10,17^ (Supplementary Table S1).

#### LST retrieval

Five Landsat 8 images (collection 2 level 2) were selected for deriving LST data. LST data are widely used as a source of temperature indicators due to their accessibility and comprehensive spatial coverage, with Landsat being the most common source of LST data retrieval due to their high spatial resolution^30^. July is the hottest period in all the cities, and therefore, the UHI effect is presumed to be stronger, and the cooling effect of urban woodland is more significant^29^. Therefore, LST in July over five years (2017–2021) was searched for each city, and the LST data were filtered to remove scenes where cloud cover contamination was > 10%. One scene was then selected for each city (Supplementary Table S8). However, the available LST data under clear sky in Greater London and Nanjing were only found in 2018 and 2017, respectively, when these two cities were experiencing higher than average air temperatures and lower than normal precipitation, making the local climate hotter and drier than usual. There are some null values in the LST data of Nanjing and Greater Toronto due to missing data.

The Landsat 8 collection 2 level 2 surface temperature product was derived from the collection 2 level 1 Thermal Infrared Sensors (TIRS) band^46^, which was further rescaled to get the LST data for each city. Scenes were chosen to maximise consistency, though some variability in the time of data collection was unavoidable (Supplementary Table S8). The cooling effect of urban woodland varies at different times of day, as the tree shade and evaporative strength change temporally^4^. Therefore, the LST data were further modelled to sync to the LST at 11:00 am by combining NDVI and air temperature data based on the following formulas.

1 $${LST}_{referece}={a}_{1}\times {T}_{a }+{b}_{1}$$

2 $${LST}_{11:00am}=a\times {LST}_{acquired time}+b\times NDVI+c$$

The above formulas assumed that LST was linearly correlated with air temperature, and the spatial variation of LST was linearly correlated with NDVI. The calculated LSTs based on air temperature were taken as reference, and the difference between LST_Reference_ (Eq. (1)) and LST_11:00am_ (Eq. (2)) was treated as the error. The parameters a, b, and c were calibrated by minimizing the RMSE, and then the LSTs at 11:00 am were modelled. Based on the LST results, a hot spot analysis was further applied to identify significant clusters of high and low temperatures across each city.

#### LCZ classification

The Local Climate Zone (LCZ) maps at level 0 for the five case study areas (Fig. 6) were generated by using the World Urban Dataset and Access Portal Tools (WUDAPT) protocol. The WUDAPT protocol provides an online platform with a supervised machine-learning approach to generate LCZ maps. Based on Google Earth Engine, the training areas were manually created as training areas and implemented into the practical workflow from WUDAPT to process the LCZ classification. The LCZ maps were at 100 m spatial resolution, and only the main LCZ classes were mapped, including six built-up LCZ classes (LCZ 1 to LCZ 6) and five land cover type LCZ classes (LCZ A, LCZ B, LCZ D, LCZ E, and LCZ G). All the LCZ results reached a relatively high weighted accuracy (over 0.85), though the overall accuracy in Greater Cairo is relatively low (0.45) (Supplementary Table S7).

### Statistical analysis

The association between LMs of urban woodland and LST was analysed using different regression models. As the 150 m study scale was identified as appropriate for the analysis, the mean LST data was calculated for each analytical unit (150 m resolution) and was used as a response variable for the five study areas, while all the 6 LMs at 150 m scale were the predictor variables.

The ordinary least square (OLS) model and geographical weighted regression (GWR) were developed for the five cities to compare the association between urban woodland composition and configuration and LST in different regional climates. The OLS model examined the ‘global’ correlation between LMs and LST at the city scale. The results of OLS models were used to quantify the significance of the correlations with Moran’s I used to examine the spatial autocorrelation of the OLS residuals. Next, GWR models were developed to quantify the local spatial variation of the correlations. OLS and GWR model performance was compared to find the better method for quantifying the correlation. After selecting the appropriate model, the effect of LMs of urban woodland on LST was compared between the cities by analysing the coefficient of each LM. The coefficients were then divided by the LCZ scheme to quantify how the effect of LMs of urban woodland spatially varied between LCZ classes. Based on the resultant coefficients, the association between LMs and LST was defined as the change in degrees of LST per unit change of LMs (for PLAND). Additionally, the standardised coefficients were used to evaluate the relative importance of spatial patterns of urban woodland on LST^16,17^. The standardised coefficients were calculated using the standardised deviation of the variables and the mean coefficients from the GWR model. The coefficient and standardised coefficient of model results were divided into each LCZ to suggest what kind of spatial patterns of urban woodland may perform cooling with the highest effectiveness in each LCZ and how these spatial patterns vary across cities. The number of patches and the patch cohesion index were excluded in the GWR model in Greater Toronto due to the local multicollinearity.

## Supplementary Information

Supplementary Information.

## Data Availability

The datasets generated and/or analysed during the current study are not publicly available due to data are currently embargoed pending a PhD examination but are available from the corresponding author on reasonable request.
