# Supplementary material for: Spatial variations in urban woodland cooling between background climates
Source: Sci Rep. 2025 Jan 25;15:3213. doi: 10.1038/s41598-024-85059-8 (PMC11763262; doi:10.1038/s41598-024-85059-8)
Supplement: Supplementary file 1 — Supplementary Information. [file 41598_2024_85059_MOESM1_ESM.pdf]

Spatial variations in urban woodland cooling between background climates

Liu, J.*, Dennis, M., and Lindley, S. J.

Department of Geography, School of Environment, Education and Development, The University of Manchester, Arthur Lewis Building, Oxford Road, Manchester M13 9PL, UK

*Corresponding Author

Email: Jianyi.liu@postgrad.manchester.ac.uk Address: Department of Geography, School of Environment, Education and Development, The university of Manchester, Arthur Lewis Building, Oxford, Manchester M13 9PL, UK

Landscape metrics and study scale selection

The appropriate landscape metrics (LMs) and study scale for analysing the association between urban woodland spatial composition and configuration and Land Surface Temperature (LST) were identified by comparing the different model performances and results based on the Greater London case.

LMs selection

Firstly, 13 class-level LMs were selected, which were commonly used in previous studies to describe spatial patterns of urban woodland[1][2] (Supplementary Table S1). To minimise the data redundancy and multicollinearity of LMs, all the LMs were analysed as independent variables, and the mean LST was the dependent variable in multilinear regression models.

Secondly, it was identified that all 13 LMs are significantly correlated (p-value<0.05) with mean LST at finer scales (90m, 120m, and 150m), while at coarser scales, some LMs are insignificant. Thus, only variables at finer scales are used to evaluate the multicollinearity.

Thirdly, less data redundancy and multicollinearity were defined as the Variance Inflation Factors (VIF) derived from the Ordinary Least (OLS) model is less than 7.5. LMs associated with VIF values larger than 7.5 were removed one by one based on Pearson's correlation matrix (Supplementary Figure S1) and the relative contribution of individual LM on the LST variation (Supplementary Table S2). Specifically, Pearson's correlation matrix (Supplementary Figure S1) shows that landscape division index, mean of patch area, and largest patch index were the most closely correlated with the composition metric (PLAND) at finer scales (90m, 120m, and 150m), which were removed firstly. Then, a close correlation was found between the mean of the fractal dimension index (Frac_mn) and the mean of the shape index (Shape_mn) and between the patch cohesion index (PCI) and aggregation index (AI), respectively. The relative importance of these LMs was identified by the R^2 of the OLS models based on each LM, which showed that Shape_mn and PCI relatively contribute more to the LST variation, and thus Frac_mn and AI are removed. The VIF of the OLS model was less than 7.5 after removing these five LMs.

Lastly, after the final check, the patch density was removed due to the collinearity between the number of patches and patch density at the same study scale, which would inhibit the

GWR model due to the local multicollinearity after the primary test. The mean of perimeter area ratio (Para_mn) and the Shape_mn describe the shape complexity of the urban woodland. Although the relative contribution of Para_mn is higher than that of Shape_mn (Supplementary Table S2), the Para_mn was removed because it was scale-dependent[3], which may lead to uncertainties in the model results. Thus, 6 of 13 LMs, including PLAND, ED, NP, PCI, ENN_MN, and Shape_mn, were selected as the appropriate LMs with less data redundancy and multicollinearity. These LMs characterise the spatial pattern of urban woodland in terms of its abundance, connectedness, aggregation, and shape complexity.

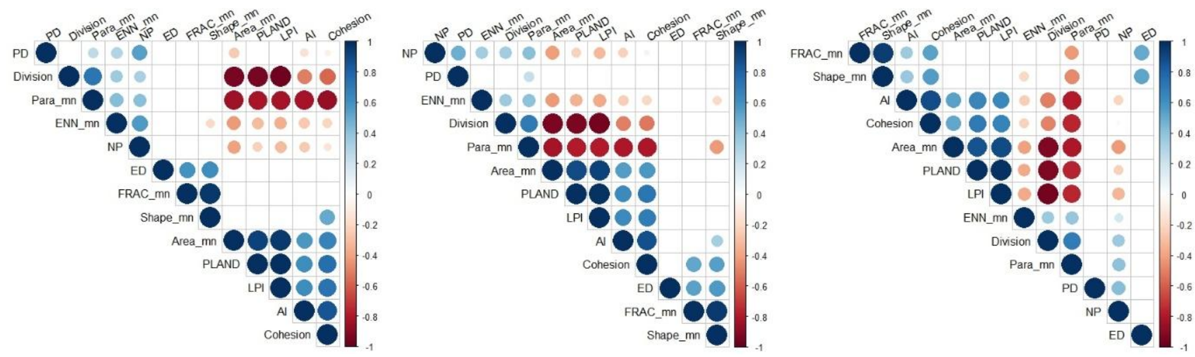

Supplementary Figure S1. The Pearson's correlation matrix (90m, 120m, and 150m scale are shown from left to right, respectively)

Scale effect and study scale selection

Due to the LST data derived from Landsat 8 collection 2 level 2 surface temperature product being resampled to 30m resolution[5], six study scales (90m, 120m, 150m, 180m, 210m, and 240m) with a 30m interval were used to test the scale effect and to select the appropriate one for analysing the association between urban woodland spatial composition and configuration and LST. These study scales were widely applied in the previous studies[6][7][8][9].

The scale effect on the association between urban woodland spatial composition and configuration and LST was identified by comparing the coefficients of the OLS and GWR models at different study scales (Supplementary Table S3). For the results of both OLS and GWR models, the coefficient of PLAND increases at coarser study scales. In comparison, when the study scales get coarser for the OLS and GWR models, the positive coefficients of edge density decrease and the negative coefficients of ENN_mn become positive. For the number of patches and the mean of the shape index, the magnitude of the coefficients decreases and even turns positive at coarser study scales.

Therefore, it is necessary to identify the appropriate study scale for analysing the association between urban woodland spatial composition and configuration and LST, and the appropriate study scale was identified based on the model performance. Specifically, the OLS model performs best at the 150m scale, and both coarser and finer scales lower the goodness of fit and increase residuals (Supplementary Figure S2a). The GWR model performs better at finer scales. However, the improvement is minimal, and the complexity of the model increases rapidly when the scale is finer than 150m, as shown in the AICc (Akaike's Information Criterion) (Supplementary Figure S2b). Finer study scales may result in highly varied and localised results[10]. To balance simplicity against complexity and balance between overfitting and underfitting[11], the 150m scale is chosen for further analysis. It accurately depicts the association between LMs of urban woodland and LSTs with relatively low complexity.

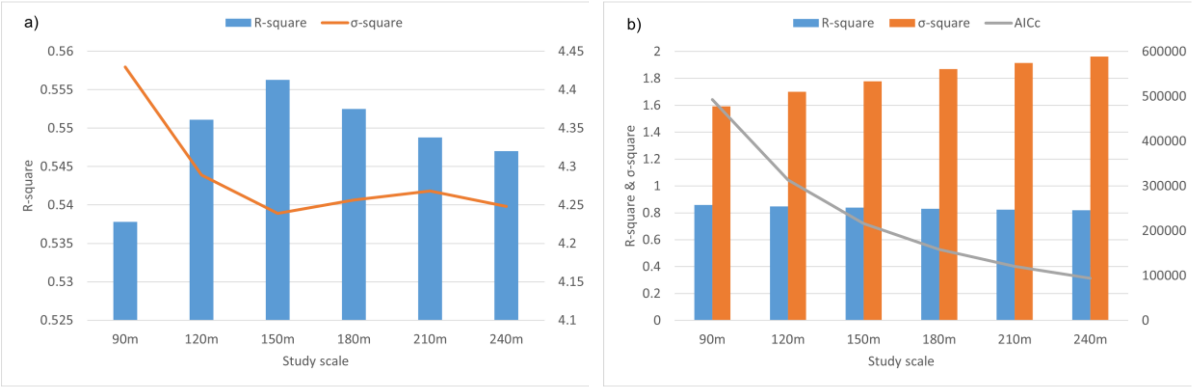

Supplementary Figure S2. The performance of models across study scales. a) Performance of the OLS models; b) Performance of the GWR models.

Supplementary Table S1. The landscape metrics[3][4]

Attribute	Landscape Metric	Units	Formular	Description
Abundance	Percentage of landscape of class (PLAND)	Percentage	$PLAND = \frac{\sum_{j=1}^{n} a_{ij}}{A} \times 100$	Refers to the percentage of landscape belonging to urban woodland.
Aggregation or fragmentation	Aggregation index (AI)	Percent	$AI = \left(\frac{g_{ii}}{max - g_{ii}}\right) \times 100$	This matrix calculates whether the land cover is aggregated.
	Number of patches (NP)	None	$NP = N$	The number of patches in a basic unit
	Mean of Euclidean nearest-neighbour distance (ENN_mn)	Meters	$ENN_{MN} = mean(ENN[patch_{ij}])$	Refer to the nearest neighbouring patch of the same class, with lower value indicating more aggregated.
	Landscape division index (Division)	Proportion	$DIVISION = (1 - \sum_{j=1}^{n} (\frac{a_{ij}}{A})^2)$	The probability that two randomly selected cells are not located in the same patch.
	Edge density (ED)	Meters per hectare	$ED = \frac{\sum_{k=1}^{m} e_{ik}}{A} \times 10000$	Aggregation of the same class will result in a low ED.
Connectivity	Patch cohesion index (PCI)	Percent	$COHESION = 1 - (\frac{\sum_{j-1}^{n} p_{ij}}{\sum_{j=1}^{n} p_{ij}\sqrt{a_{ij}}}) \times (1 - \frac{1}{\sqrt{z}})^{-1} \times 100$	Describes the connectedness of patches.
Density	Patch density (PD)	Number per 100 hectares	$PD = \frac{N}{A} \times 10000 \times 100$	The density of patches in a basic unit.

Size	Largest patch index (LPI)	Percentage	$LPI = \frac{max_{j=1}^{n}(a_{ij})}{A} \times 100$	The percentage of landscape covered by the corresponding largest patch.
	Mean of the patch area (Area_mn)	Hectares	$AREA_{MN} = mean(AREA[patch_{ij}])$	Summary mean of urban woodland area.
Shape complexity	Mean of the shape index (Shape_mn)	None	$SHAPE_{MN} = mean(SHAPE[patch_{ij}]])$	Describe the complexity of the shape by its perimeter.
	Mean of the perimeter-area ratio (Para_mn)	None	$PARA_{MN} = mean(PARA[patch_{ij}])$	Describes the patch complexity.
	Mean of the fractal dimension index (Frac_mn)	None	$FRAC_{MN} = mean(FRAC[patch_{ij}])$	This matrix describe the patch complexity by its perimeter and patch area.

a_{ij}: The area of each patch; e_{ik}: Total edge length in meters; A: The total landscape area in square meters; g_{ii}: number of like adjacencies based on the single-count methods; max-g_{ii}: the class wise maximum number of like adjacencies of class i; p_{ij}: The perimeter in meters; Z: The number of cells; N: The number of patches

Supplementary Table S2. The R^2 of the OLS model based on individual LM

Scale	AI	PCI	Shape_mn	Frac_mn	ENN_mn	NP	Para_mn	PD	ED
90m	0.20	0.29	0.01	Lower than 0.01	0.04	0.02	0.34	0.01	0.01
120m	0.23	0.29	0.02	0.01	0.07	0.03	0.35	0.01	0.01
150m	0.26	0.30	0.04	0.01	0.08	0.04	0.36	0.01	0.01

Supplementary Table S3. The coefficients of the OLS models and the mean coefficients of the GWR models

	PLAND	ED	PCI	NP	ENN_mn	Shape_mn
OLS model results						
90m	-0.0712	0.0066	-0.0117	-0.5016	-0.0003	-1.3171
120m	-0.0776	0.0054	-0.0130	-0.2360	0.0013	-0.6843
150m	-0.0801	0.0043	-0.0150	-0.0849	0.0027	-0.2508
180m	-0.0827	0.0037	-0.0138	-0.0149	0.0035	-0.2312
210m	-0.0822	0.0030	-0.0148	0.0331	0.0054	-0.0275
240m	-0.0835	0.0026	-0.0136	0.0479	0.0059	0.0033
GWR model results						
90m	-0.0522	0.0044	-0.0077	-0.3682	-0.0026	-1.0141
120m	-0.0646	0.0040	-0.0081	-0.2256	-0.0019	-0.7098
150m	-0.0718	0.0033	-0.0089	-0.1195	-0.0012	-0.4002
180m	-0.0790	0.0033	-0.0080	-0.0710	-0.0005	-0.4851
210m	-0.0822	0.0030	-0.0090	-0.0342	0.0001	-0.3637
240m	-0.0854	0.0029	-0.0083	-0.0140	0.0010	-0.3929

Supplementary tables and figures

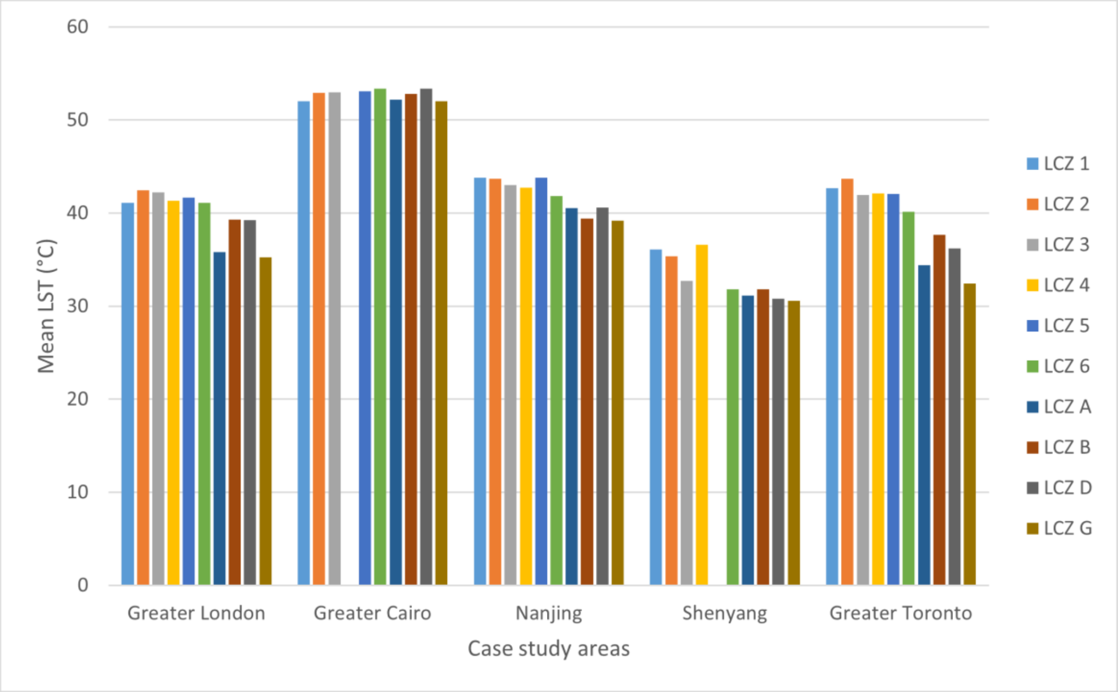

Supplementary Figure S3. The mean LST in each LCZ class across cities.

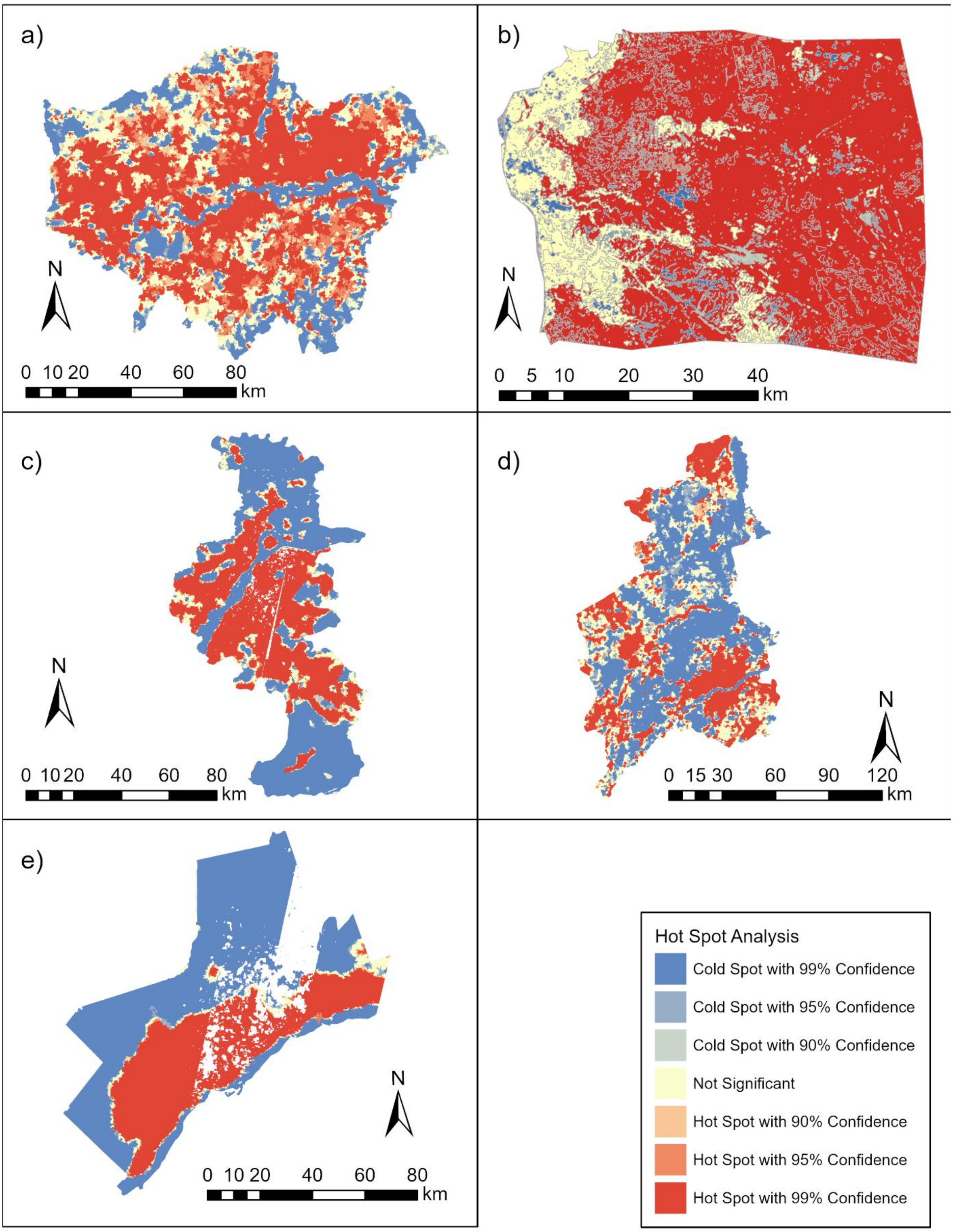

Supplementary Figure S4. Hot spot analysis of LST across case study areas. a) to e) presents the LST in Greater London, Greater Cairo, Nanjing, Shenyang, and Greater Toronto, respectively. ArcGIS Pro, Version 3.0.2 (https://www.esri.com/en-us/arcgis/products/arcgis-pro/overview)

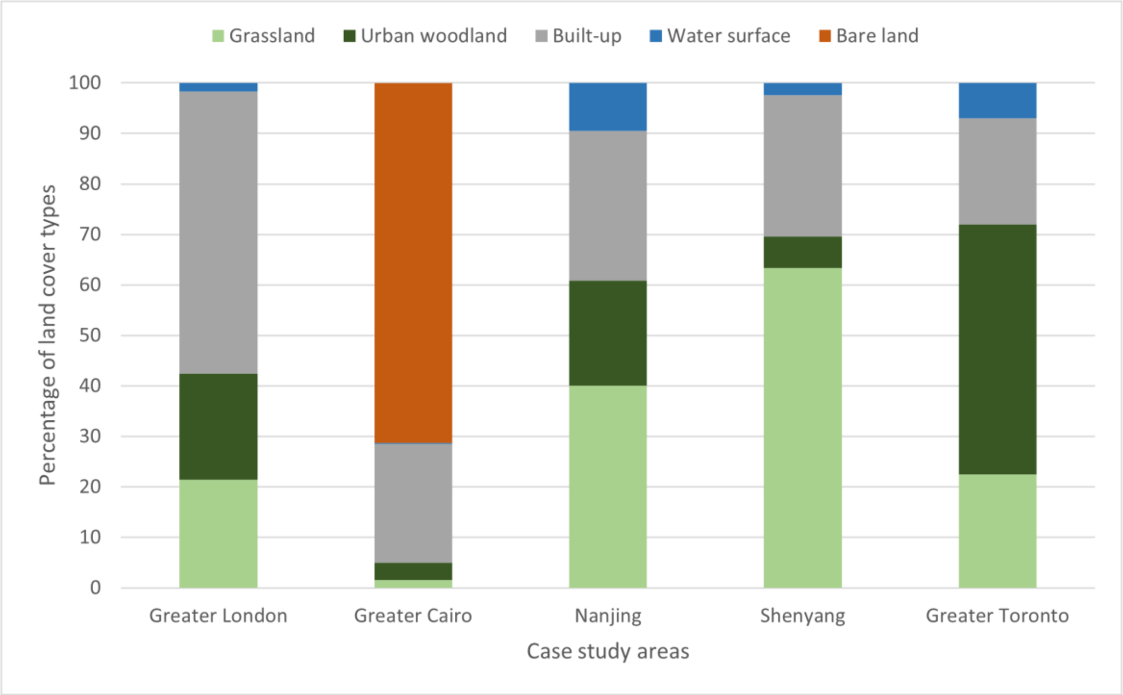

Supplementary Figure S5. The percentage of each land cover type across case study areas

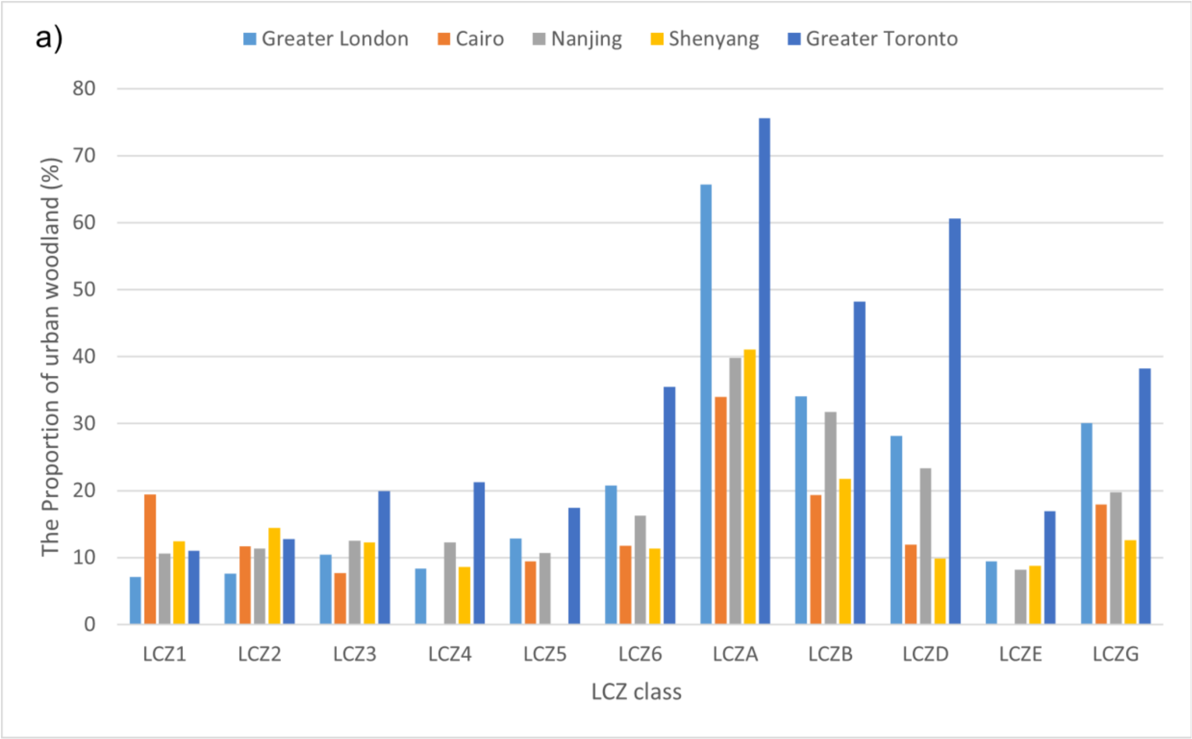

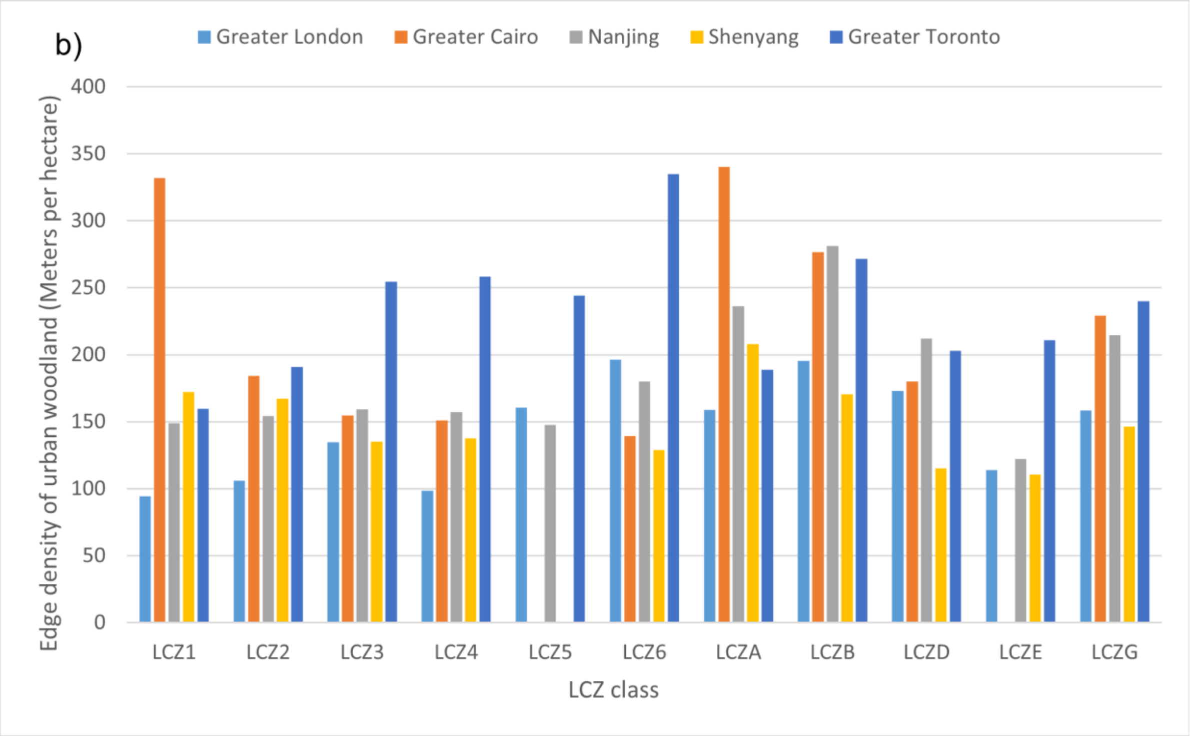

Supplementary Figure S6. The characteristics of urban woodland across LCZ classes among five case studies.a) The percent coverage of urban woodland; b) the edge density of urban woodland.

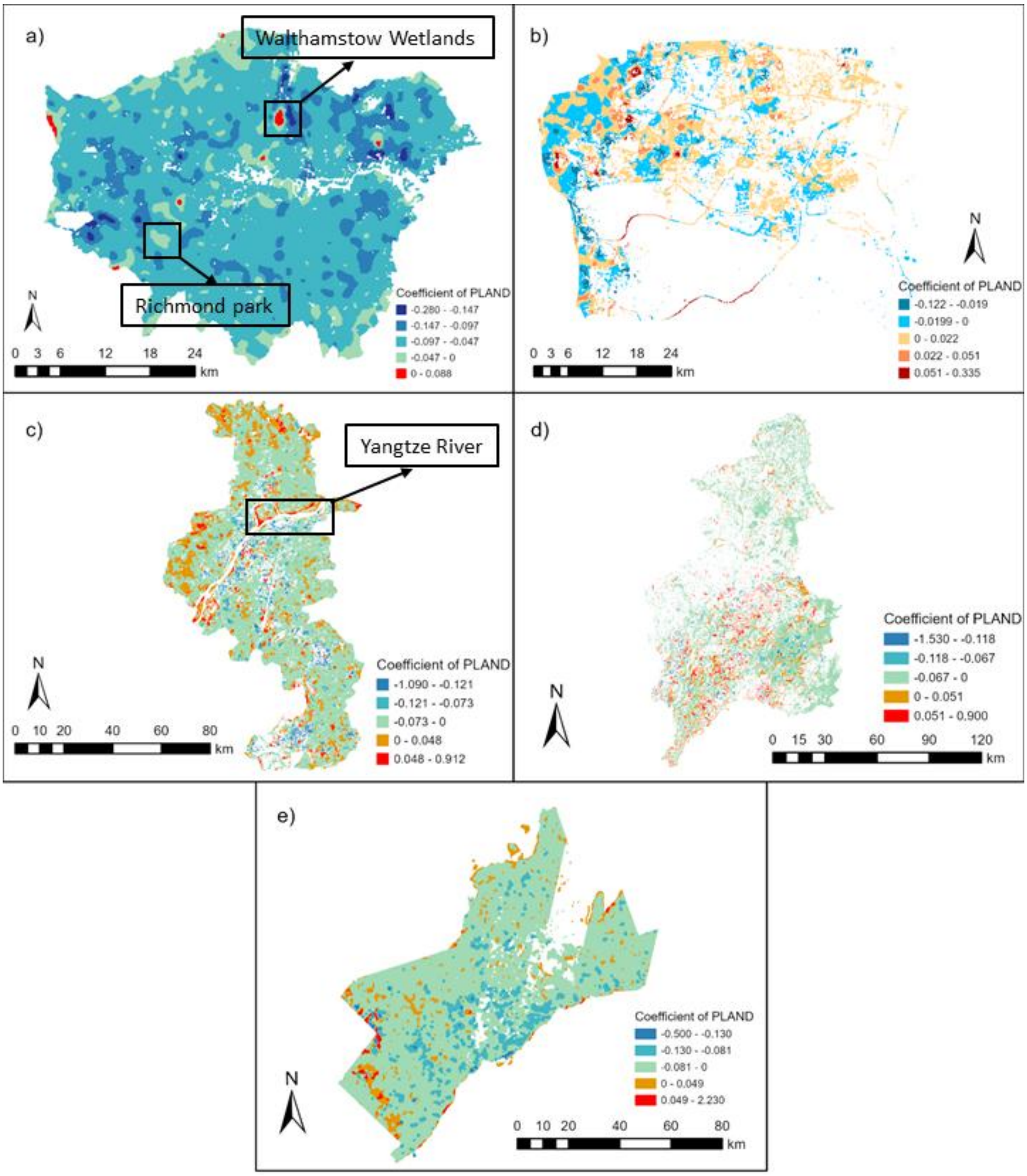

Supplementary Figure S7. The distribution of PLAND coefficient of the case study areas. a) to e) presents the spatial distribution of PLAND coefficient in Greater London, Greater Cairo, Nanjing, Shenyang, and Greater Toronto, respectively. ArcGIS Pro, Version 3.0.2 (https://www.esri.com/en-us/arcgis/products/arcgis-pro/overview)

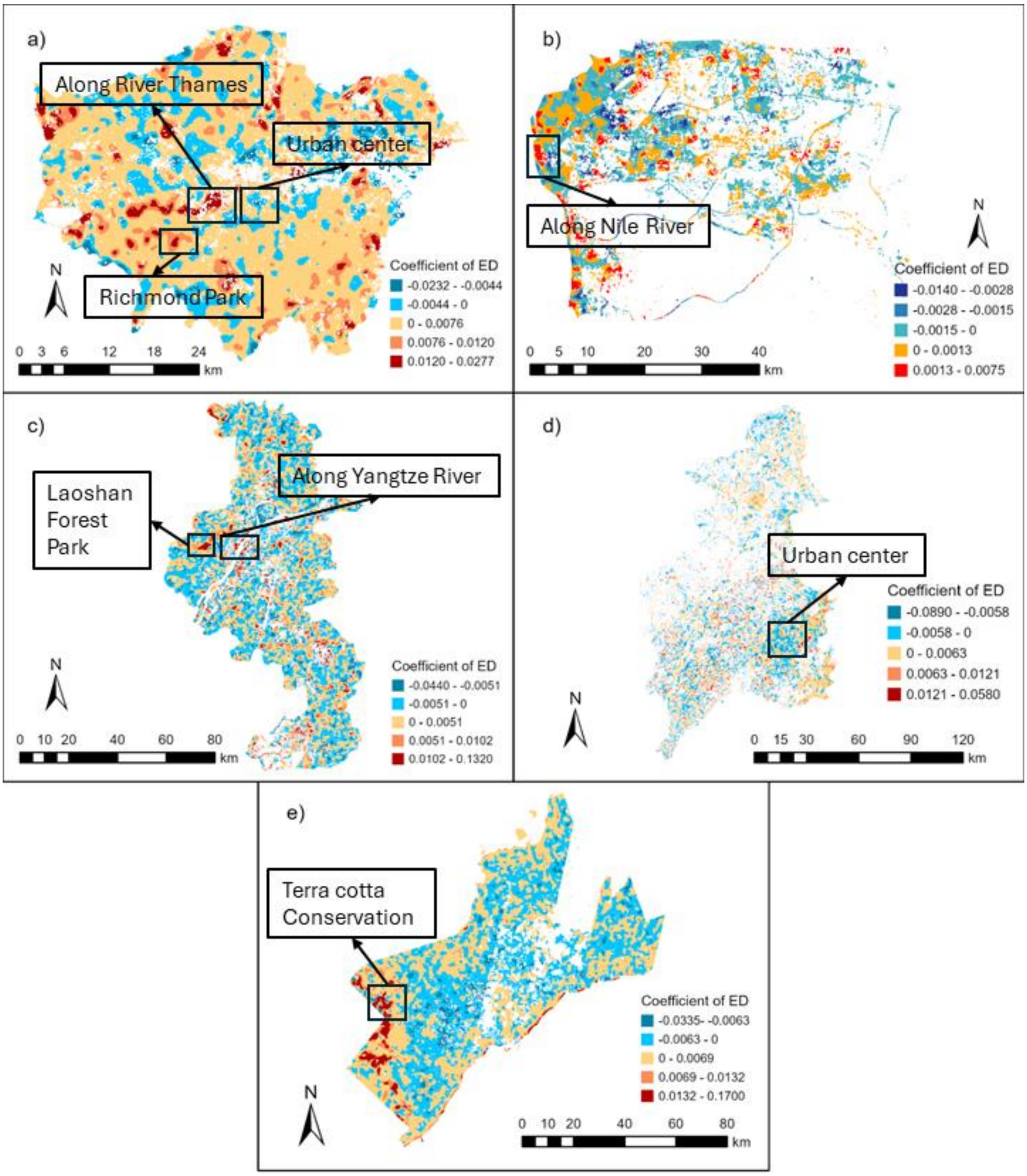

Supplementary Figure S8. The distribution of ED coefficients of the case study areas. a) to e) presents the spatial distribution of ED coefficient in Greater London, Greater Cairo, Nanjing, Shenyang, and Greater Toronto, respectively. ArcGIS Pro, Version 3.0.2 (https://www.esri.com/en-us/arcgis/products/arcgis-pro/overview)

Supplementary Table S4. The coefficients of the GWR models

	Greater London	Greater Cairo	Nanjing	Shenyang	Greater Toronto
GWR coefficients of PLAND					
Lower quartile	-0.0869	-0.0045	-0.0459	-0.0393	-0.0620
Upper quartile	-0.0569	0.0081	-0.0041	-0.0008	-0.0162
Mean	-0.0718	0.0023	-0.0271	-0.0169	-0.0359
Median	-0.0713	0.0013	-0.0239	-0.0230	-0.0368
GWR coefficients of ED					
Lower quartile	0.0005	-0.0008	-0.0017	-0.0019	-0.0015
Upper quartile	0.0057	0.0004	0.0030	0.0034	0.0028
Mean	0.0033	-0.0002	0.0009	0.0006	0.0006
Median	0.0028	-0.0002	0.0005	0.0008	0.0013
GWR coefficients of ENN_mn					
Lower quartile	-0.0056	-0.0012	-0.0080	-0.0051	-0.0130
Upper quartile	0.0029	0.0004	0.0049	0.0042	0.0055
Mean	-0.0012	-0.0002	-0.0009	-0.0002	-0.0032
Median	-0.0013	-0.0004	-0.0016	-0.0006	-0.0034
GWR coefficients of Shape_mn					
Lower quartile	-0.9491	-0.1326	-0.7926	-0.8067	-0.8911
Upper quartile	0.2340	0.1149	0.3546	0.3224	0.3608
Mean	-0.4002	-0.0081	-0.2229	-0.2360	-0.2994
Median	-0.3371	-0.0128	-0.1956	-0.2320	-0.2361
GWR coefficients of PCI					
Lower quartile	-0.0151	-0.0008	-0.0166	-0.0098	
Upper quartile	-0.0002	0.0019	0.0038	0.0057	
Mean	-0.0089	0.0004	-0.0078	-0.0024	
Median	-0.0064	0.0005	-0.0059	-0.0019	
GWR coefficients of NP					
Lower quartile	-0.2154	-0.0333	-0.1526	-0.1674	
Upper quartile	-0.0386	0.0095	0.0926	0.0656	

Mean	-0.1195	-0.0118	-0.0250	-0.0475
Median	-0.1339	-0.0116	-0.0316	-0.0534

Supplementary Table S5. The mean of standardised coefficients of the GWR models

	Greater London	Greater Cairo	Nanjing	Shenyang	Greater Toronto
PLAND	-0.6054	0.0573	-0.2315	-0.1279	-0.31
ED	0.1278	-0.0472	0.0383	0.0261	0.0549
NP	-0.0841	-0.054	-0.0166	-0.0288	
PCI	-0.0834	0.0222	-0.0771	-0.0278	
ENN_mn	-0.01	-0.0075	-0.0068	-0.002	-0.0142
Shape_mn	-0.0342	-0.0039	-0.0255	-0.0255	-0.0261

Supplementary Table S6. The accuracy of the land cover classification (%)

Overall accuracy				
Greater London	Greater Cairo	Nanjing	Shenyang	Greater Toronto
95.18	92.35	89.57	95.87	91.17

User's accuracy				
	Grassland	Tree Cover	Built-up area	Water surface
Greater London	95.99	91.78	95.25	99.07
Greater Cairo	84.92	78.33	93.47	97.20
Nanjing	92.69	82.90	93.30	85.51
Shenyang	96.96	93.58	95.72	94.90
Greater Toronto	84.65	92.39	95.52	90.41

Producer's accuracy				
	Grassland	Tree Cover	Built-up area	Water surface
Greater London	86.58	91.79	98.79	1
Greater Cairo	63.19	70.47	98.43	43.58
Nanjing	90.77	93.83	84.14	90.10
Shenyang	95.86	86.76	97.57	93.87
Greater Toronto	86.22	94.08	88.91	93.89

Supplementary Table S7. The accuracy metrics of the LCZ mapping results

	Greater London	Greater Cairo	Nanjing	Shenyang	Greater Toronto
OA	0.76	0.45	0.75	0.73	0.61
OA_U	0.79	0.45	0.51	0.67	0.70
OA_{bu}	0.98	0.88	0.98	0.96	0.89
OA_w	0.92	0.85	0.92	0.93	0.88

Supplementary Table S8. The sense date and time of day of LST across case study cities.

	Greater London	Greater Cario	Nanjing	Shenyang	Greater Toronto
Date of collection	15/07/2018	16/07/2018	21/07/2017	04/07/2019	02/07/2020
Time of day	10:52	8:22	14:36	14:28	16:03

Reference

[1] Liu, K., Li, X., Wang, S. and Gao, X. Assessing the effects of urban green landscape on urban thermal environment dynamic in a semiarid city by integrated use of airborne data, satellite imagery and land surface model. *International Journal of Applied Earth Observations and Geoinfomation,* 107 (2022).

[2] Zhou, W., Wang, J. and Cadenasso, M. L. Effects of the spatial configuration of trees on urban heat mitigation: A comparative study. *Remote Sensing of Environment*, 195, 1-12 (2017).

[3] McGarigal, K., Cushman, S. A. and Ene, E. FRAGSTATS v4: Spatial Pattern Analysis Program for Categorical Maps. Computer software program produced by the authors https://www.fragstats.org (2023).

[4] Hesselbarth, M. H. K., Sciaini, M, With, K. A., Wiegand, K. and Nowosad, J. Landscapemetrics: an open-source R tool to calculate landscape metrics. *Ecography*, 42(10), 1648-1657 (2019).

[5] Earth Resources Observation and Science (EROS) Center. Landsat 8-9 Operational Land Imager / Thermal Infrared Sensor Level-2, Collection 2 [dataset]. U.S. Geological Survey. https://doi.org/10.5066/P9OGBGM6 (2020).

[6] Bao, T., Li, X., Zhang, J., Zhang, Y. and Tian, S. Assessing the distribution of urban green spaces and its anisotropic cooling distance on urban heat island pattern in Baotou, China. *ISPRS Internation Journal of Geo-information*, 5(2) (2016).

[7] Rhee, J., Park, S. and Lu, Z. Relationship between land cover patterns and surface temperature in urban areas. *Giscience and Remote Sensing*, 51(5), 521-536 (2014).

[8] Myint, S. W., Wentz, E. A., Brazel, A. J. and Quattrochi, D. A. The impact of distinct anthropogenic and vegetation features on urban warming. *Landscape Ecology*, 28(5), 959-978 (2013).

[9] Kong, F., Yin, H., James, P., Hutyra, L. & He, H. Effects of spatial pattern of greenspace on urban cooling in a large metropolitan area of eastern China. Landscape and Urban Planning, 128, 35-47 (2014).

[10] Chien, Y. M. C., Carver, S. and Comber, A. Using geographically weighted models to explore how crowdsourced landscape perceptions relate to landscape physical characteristics. Landscape and Urban Planning, 203 (2020).

[11] Claeskens, G. and Hjort, N. L. Model selection: data examples and introduction in Model selection and model averaging 1-21 (Cambridge Series in Statistical and Probabilistic Mathematics, 2008).
